# The importance of vitamin d metabolism as a potential prophylactic, immunoregulatory and neuroprotective treatment for COVID-19

**DOI:** 10.1186/s12967-020-02488-5

**Published:** 2020-08-26

**Authors:** Yi Xu, David J. Baylink, Chien-Shing Chen, Mark E. Reeves, Jeffrey Xiao, Curtis Lacy, Eric Lau, Huynh Cao

**Affiliations:** 1 Division of Hematology and Oncology, Department of Medicine, Loma Linda University, Loma Linda, California USA; 2 Division of Regenerative Medicine, Department of Medicine, Loma Linda University, Loma Linda, California USA; 3 Loma Linda University Cancer Center, Loma Linda, California USA

**Keywords:** Coronavirus, COVID-19, Vitamin D, 1,25(OH)_2_D_3_, 25(OH)D, Infection, Immunomodulation, Extra-renal, Metabolism, NF-kB, IL-6, TNF, NGF

## Abstract

The coronavirus disease 2019 (COVID-19) pandemic has led to a declaration of a Public Health Emergency of International Concern by the World Health Organization. As of May 18, 2020, there have been more than 4.7 million cases and over 316,000 deaths worldwide. COVID-19 is caused by a highly infectious novel coronavirus known as severe acute respiratory syndrome coronavirus-2 (SARS-CoV-2), leading to an acute infectious disease with mild-to-severe clinical symptoms such as flu-like symptoms, fever, headache, dry cough, muscle pain, loss of smell and taste, increased shortness of breath, bilateral viral pneumonia, conjunctivitis, acute respiratory distress syndromes, respiratory failure, cytokine release syndrome (CRS), sepsis, etc. While physicians and scientists have yet to discover a treatment, it is imperative that we urgently address 2 questions: how to prevent infection in immunologically naive individuals and how to treat severe symptoms such as CRS, acute respiratory failure, and the loss of somatosensation. Previous studies from the 1918 influenza pandemic have suggested vitamin D’s non-classical role in reducing lethal pneumonia and case fatality rates. Recent clinical trials also reported that vitamin D supplementation can reduce incidence of acute respiratory infection and the severity of respiratory tract diseases in adults and children. According to our literature search, there are no similar findings of clinical trials that have been published as of July 1st, 2020, in relation to the supplementation of vitamin D in the potential prevention and treatment for COVID-19. In this review, we summarize the potential role of vitamin D extra-renal metabolism in the prevention and treatment of the SARS-CoV-2 infection, helping to bring us slightly closer to fulfilling that goal. We will focus on 3 major topics here:

1. Vitamin D might aid in preventing SARS-CoV-2 infection:
  Vitamin D: Overview of Renal and Extra-renal metabolism and regulation.
  Vitamin D: Overview of molecular mechanism and multifaceted functions beyond skeletal homeostasis.
  Vitamin D: Overview of local immunomodulation in human infectious diseases.
    Anti-viral infection.
    Anti-malaria and anti-systemic lupus erythematosus (SLE).
2. Vitamin D might act as a strong immunosuppressant inhibiting cytokine release syndrome in COVID-19:
  Vitamin D: Suppression of key pro-inflammatory pathways including nuclear factor kappa B (NF-kB), interleukin-6 (IL-6), and tumor necrosis factor (TNF).
3. Vitamin D might prevent loss of neural sensation in COVID-19 by stimulating expression of neurotrophins like Nerve Growth Factor (NGF):
  Vitamin D: Induction of key neurotrophic factors..

## Background

COVID-19 is an infectious disease caused by SARS-CoV-2, a newly discovered coronavirus that primarily spreads between people during close contact and through respiratory droplets when infected individuals cough, sneeze, or talk [1–3]. Furthermore, infection might occur from the touching of a contaminated surface and subsequent contact with the face [3]. SARS-CoV-2 is an enveloped and single positive-stranded RNA virus (~ 30 kb in length) with a nucleocapsid, which undergoes endocytosis or membrane fusion to enter the infected cells and can cause respiratory, enteric, hepatic, and neurological diseases in different species including humans [4]. Mechanistically, SARS-CoV-2 has spike (S) glycoproteins comprised of two functional subunits called the S1 protein which binds to the host cell receptor and the S2 protein which promotes fusion of the viral and cellular membranes [5]. Angiotensin Converting Enzyme II Receptor (ACE2) has been identified as a functional receptor for SARS-CoV-2 entry into the cell [6–8], and ACE2 expression is high in the lung, heart, ileum, kidney and bladder [9]. At this time, there are no specific vaccines or treatments for COVID-19, and older adults with underlying comorbidities are at higher risk for severe illness [10]. Thus, important information for most people is to know how to enhance their immune system to prevent SARS-CoV-2 infection or control the severity of disease progression to avoid the next waves of the deadly COVID-19 pandemic [11].

The last century’s influenza pandemic (1918–1919) claimed between 40 and 50 million lives [12]. A substantial proportion of deaths were found to occur greater than 2 weeks after symptom onset, suspected to be caused by cytokine storms [13]. Unfortunately, the second and third waves of the 1918–1919 pandemic were much more deadly than the first one because the H1N1 virus had mutated to a deadlier form and spread worldwide faster [14]. Analyzing data during the 1918–1919 pandemic revealed an inverse association between solar ultraviolet B (UVB) irradiance and case fatality rate, suggesting that vitamin D might have played a role in reducing the development of pneumonia and improving case fatality rates [15]. The lowest pneumonia and case-fatality rates were found in the region with the highest solar UVB irradiance and lowest latitude in San Antonio, Texas, while the highest rates were in New London, Connecticut, which had the lowest UVB irradiance and highest latitude. It seems that a similar phenomenon is occurring in the first wave of the current COVID-19 pandemic [16].

Vitamin D is a group of fat-soluble steroids, and the most common forms of vitamin D supplementation are cholecalciferol (Vitamin D_3_) and ergocalciferol (Vitamin D_2_), precursors of 1,25(OH)_2_D_3_ (the active form of vitamin D) [17]. New developments in measurement of vitamin D metabolites for both clinical diagnosis and research practice has greatly advanced our understanding of Vitamin D’s role in human health in the last decade [18]. Vitamin D is involved in essential biological roles (classical) including bone metabolism, calcium and phosphorus homeostasis, and recently discovered roles (non-classical) involving immunomodulation, lung and muscle function, cardiovascular health, and infectious disease prevention [17, 19, 20]. In COVID-19 patients, type-II alveolar epithelial cells (pneumocytes) are the primary target of SARS-CoV-2, and their impairment decreases the surfactant level and increases the risk of acute respiratory distress syndrome (ARDS) [21, 22]. Vitamin D has been reported to reduce apoptosis of pneumocytes and stimulate surfactant synthesis in these cells to prevent severe lung injuries such as ARDS [23, 24]. Vitamin D sufficiency is widely defined as a serum 25-hydroxyvitamin D (25(OH)D) level greater than or equal to 30 ng/ml (75 nmol/L), while insufficiency is defined as 20 to 30 ng/ml (50–75 nmol/L), and deficiency is a level lower than 20 ng/ml (50 nmol/L) [25]. A low level of vitamin D is common in the elderly and has been associated with all-cause mortality including sepsis, and that supplementation of vitamin D could significantly reduce overall mortality [26, 27]. Besides geographic and climate factors, vitamin D deficiency is also more prevalent among individuals with darker skin than those with a lighter skin color due to the fact that heavy pigmentation reduces vitamin D production in the skin [28]. Furthermore, an additional burden of obesity or chronic diseases make them much more susceptible to H1N1 or COVID-19. Previous clinical trials reported that vitamin D supplementation can reduce incidence of acute respiratory infection and the severity of respiratory tract diseases in adults and children [29, 30]. The purpose of this review is to provide an overview of vitamin D’s newly discovered non-classical functional roles in immunomodulation to prevent viral infection, inhibit CRS, and reveal the importance of understanding vitamin D extra-renal metabolism in pursuit of alternative benefits of vitamin D supplementation. In addition, we hypothesize that maintaining a healthy baseline level of vitamin D may lead to improved outcomes in COVID-19 patients, including many minorities.

## Main text

### Vitamin D might aid in preventing SARS-CoV-2 infection through immunomodulation

#### Overview of vitamin D Renal and Extra-renal metabolism and regulation (Fig. 1)

In humans, the main source of vitamin D is derived from dermal synthesis with a small proportion coming from food sources [19]. To become biologically active, vitamin D undergoes serial enzymatic conversions through hydroxylation to initially convert to 25(OH)D, and then primarily in the kidneys, the enzyme 1α-hydroxylase (CYP27B1) produces 1α,25-dihydroxyvitamin D3 (1,25(OH)_2_D_3_), the active form of vitamin D. Systemically, vitamin D plays a critical role in calcium homeostasis and bone metabolism by binding the vitamin D receptor (VDR) to form vitamin D response element (VDRE) to regulate expression of the down-stream genes of vitamin D [19]. Parathyroid hormone (PTH) is the main hormone in calcium homeostasis, regulating vitamin D levels through the regulation of renal CYP27B1.

**Fig. 1:**
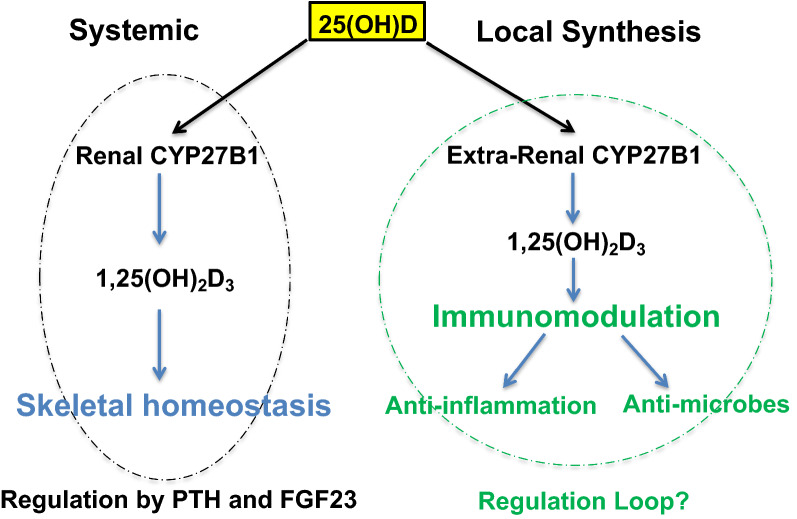
Schematic overview of vitamin D metabolism and functions. Conversion of pro-hormone 25(OH)D to 1,25(OH)_2_D_3_ occurs primarily in kidneys, which is catalyzed by the enzyme CYP27B1 and released systemically to maintain skeletal homeostasis including calcium and phosphate metabolism. PTH is the main hormone in calcium homeostasis, regulating systemic calcium levels through its regulation of renal CYP27B1. Expression of CYP27B1 in extra-renal tissues has been demonstrated, which supports local synthesis of 1,25(OH)_2_D_3_ to act as immunosuppressant during the inflammation or invasion of microbe. The regulatory loop of extra-renal CYP27B1 and local synthesis of 1,25(OH)_2_D_3_ is not fully understood

Accumulating evidence demonstrates that extra-renal synthesis of 1,25(OH)_2_D_3_ is critical for the immunomodulatory function of vitamin D in local tissues [31]. However, molecular and cellular mechanisms underpinning the regulation of local synthesis of 1,25(OH)_2_D_3_ and expression of CYP27B1 are not fully understood because of the lack of tools to acutely measure 1,25(OH)_2_D_3_ levels in their microenvironment under physiological or pathogenic conditions and different regulatory mechanisms of extra-renal CYP27B1 between tissues [32].

#### Molecular mechanism and multifaceted functions of vitamin D beyond the bone

Many cellular and molecular targets of vitamin D have been identified in cancer studies during the past decades [19]. Mechanistically, a number of vitamin D–induced signaling pathways involving PI3K, PKC, JNK, and ERK have been elucidated to play critical roles in cell differentiation. Being a powerful inhibitor of cyclin-dependent kinases, a pro-apoptotic agent through upregulation of pro-apoptotic protein Bax, and a down-regulator of the anti-apoptotic protein BCL-2, vitamin D is an attractive therapeutic option for the treatment of some human diseases [8, 15]. However, genetic variants of VDR and low 25(OH)D levels were reported in patients affected by immune-regulated disorders such as autoimmunity, infectious diseases, sepsis, and cancers [33]. Furthermore, decreased levels of CYP27B1 expression in cancer patients demonstrated the importance of vitamin D’s autocrine/paracrine function in maintaining normal proliferation and differentiation of local tissues [34].

#### Immunomodulatory functions of extra-renal vitamin D metabolism in human infectious diseases

##### Vitamin D protects against pathogens by exerting important regulatory functions on both innate and adaptive immunity [35–47] (Fig. 2)

The immune system is divided into two branches: innate and adaptive immunity. The innate response is the first line of defense relying largely on mucosal barriers, monocytes, neutrophils, macrophages, and dendritic cells, which also function as antigen presenting cells that activate the B and T lymphocytes of the adaptive immune response. The expression of CYP27B1 and VDR were found in most immune cells including macrophages, dendritic cells, and activated B and T lymphocytes [31]. Emerging data highlight the activation of natural killer (NK) cells, innate immune effector cells, as not only a contributor to the resolution of SARS-CoV-2 infection, but a contributor to the cytokine storm found in ARDS as well [48]. However, multiple studies have reported impaired cellular functions of ex vivo NK cells isolated from the peripheral blood of COVID-19 patients [48, 49]. There is no available data reports of vitamin D’s effect on NK cells in COVID-19 patients so far, but previous results suggest that the decreased serum calcitriol might contribute to the diminished NK activity in patients with chronic diseases, and vitamin D supplementation could significantly increase cytotoxicity and exocytosis of NK cells [50, 51].

**Fig. 2:**
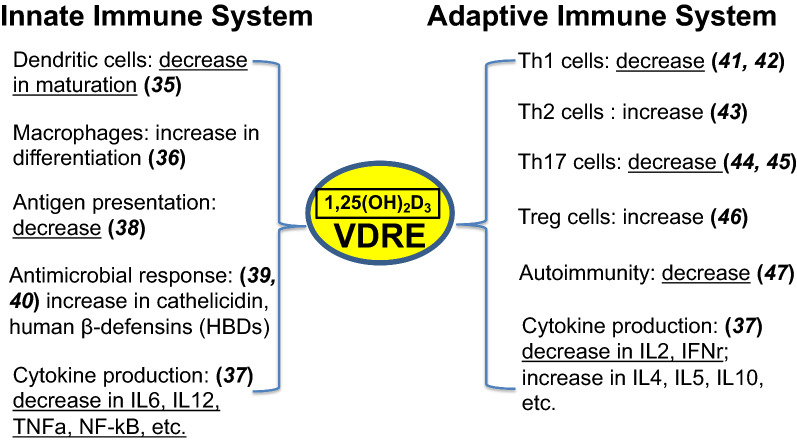
Overview of vitamin D’s immunomodulation functions. Innate immune response: Vitamin D inhibits the maturation of dendritic cells and blocks their antigen presentation to T helper cells. Also, vitamin D induces the differentiation of macrophages and exerts direct antibacterial and antiviral actions through induction of cathelicidin and defensin peptides. Adaptive immune response: Vitamin D modulates the balance of T-helper subsets by inhibiting Th1 and Th17 effector cells, inducing Th2 cells, and enhancing the development of Treg cells. Vitamin D suppresses the release of pro-inflammatory cytokines including IL2, IL6, IL12, INFr, TNFa, NF-kB, etc., from both innate and adaptive immune responses

Vitamin D is known to exert direct antibacterial and antiviral actions through cathelicidin (LL37), an antimicrobial peptide that promotes the induction of reactive oxygen species and the inhibition of phospholipids’ synthesis [52, 53]. Vitamin D also induces differentiation of macrophages and upregulates the expression of CD14 and the Toll-like receptors (TLRs) 2/4 co-receptor, stimulating the expression of CYP27B1 in macrophages while inhibiting the maturation of dendritic cells and blocking their antigen presentation that starts an adaptive response [53]. Vitamin D plays a role in enhancing the development of T regulatory (Treg) cells and balancing T-helper (Th, CD4 +) cell responses to defend against pathogens and decrease release of pro-inflammatory cytokines [53]. In summary, local vitamin D increases antimicrobial activity through modulating the innate response, balancing Th cells’ defense against pathogens, and decreasing pro-inflammatory effects of both innate and adaptive immune responses [52, 53].

##### Vitamin D has antiviral function [53–61] (Fig. 3)

Low levels of vitamin D have been reported to be involved in the pathogenesis of many infectious diseases including bacterial or viral infections of the respiratory tract such as tuberculosis and influenza, Human Immunodeficiency Virus (HIV), Epstein Barr Virus (EBV), Hepatitis C Virus (HCV), parasitic infections of the gastrointestinal tract, and systemic fungal infection [53].

**Fig. 3:**
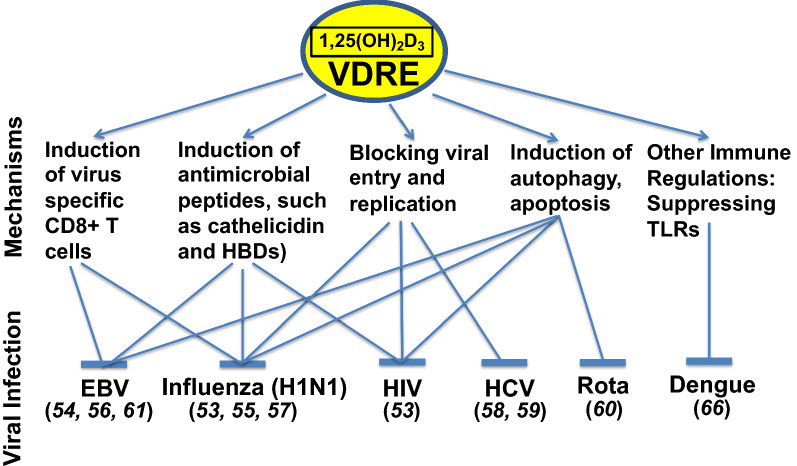
Mechanism of vitamin D’s Anti-Viral Functions. (1) Induction of virus-specific CD8 + T cells (EBV and Influenza). (2) Induction of cathelicidin and HBDs (EBV, Influenza, and HIV). (3) Blocking of viral entry and replication (Influenza, HIV, HCV). (4) Induction of autophagy and apoptosis (EBV, Influenza, HIV, Rota). (5) Suppression of Toll-like Receptors like TLR2, TLR7, and TLR9 (Dengue)

Vitamin D has been found to be a component of innate responses needed to maintain respiratory tract health. Mechanistically, acute viral respiratory tract infections have been found to upregulate CYP27B1 in respiratory epithelial cells, converting local stores of vitamin D into 1,25-dihydroxyvitamin D leading to the induction of cathelicidin [62]. This increase in 1,25(OH)_2_D_3_ then leads to downstream changes in gene expression, ultimately reducing inflammation while maintaining antiviral function [63]. Supplementation with vitamin D has been found to induce significantly elevated levels of cathelicidin, which recruits neutrophils, monocytes, and T cells to infected tissues, suppresses the activity of pathogens, and promotes clearance of respiratory pathogens by inducing apoptosis of infected epithelial cells [64]. Vitamin D is also known to improve the efficacy of interferon (IFN)-based therapy and CD8+ T cells’ responses against EBV-infected B cells [54].

TLRs play a key role in the mediation of early pathogen recognition during early stages of viral infections. TLR1/2 heterodimers can activate human macrophages and induce expression of CYP27B1 and VDR, leading to the induction of antimicrobial peptides [65]. A recent ex vivo clinical trial reported that supplementation with 4000 international units (IU)/day of vitamin D decreased dengue virus infection through modulating the innate immune response, downregulating the expression of TLR3, TLR7, TLR9, IL12, and IL8 but increasing IL10 secretion [66].

The induction of autophagy and apoptosis are new mechanisms by which vitamin D may exert its potential effects against viral infections including HIV-1, rotavirus (Rota), and kaposi sarcoma associated herpesvirus [53]. There are ongoing clinical trials of vitamin D in the prevention and treatment of pulmonary infection by utilizing its anti-microbial function and immunomodulatory role (Studies 5–9, Table 1). Our hypothesis is that vitamin D and its analogs might be considered as promising supplementary therapeutics in preventing infection of SARS-CoV-2.

**Table 1:** Ongoing clinical trials as of April 18th, 2020, of vitamin D in the prevention and treatment of human diseases

Study	Year	Disease	Title	Role	Dose of vitamin D	Status	Country	Trial ID#
1	2020–2020	COVID-19	Vitamin D on Prevention and Treatment of COVID-19	Prevention, Treatment	A single 25,000 IU	NEW: Not yet recruiting	Spain	NCT04334005
2	2020–2021	COVID-19	A study of hydroxychloroquine, Vitamin C, Vitamin D, and Zinc for the Prevention of COVID-19 infection	Prevention, treatment	Not available	New: Not yet recruiting	USA	NCT04335084
3	2020–2021	COVID-19	A study of quintuple therapy to treat COVID-19 Infection	Prevention, treatment	Not available	New: Not yet recruiting	USA	NCT04334512
4	2020–2020	COVID-19	Proflaxis using hydroxychloroquine plus vitamins-zinc during COVID-19 pandemia	Prevention, treatment	Not available	New: recruiting	Turkey	NCT04326725
5	2020–2024	Respiratory infection	Daily vitamin D for sickle-cell respiratory complications	Prevention	3333 IU daily and 100,000 IU monthly	New: Not yet recruiting	USA	NCT04170348
6	2010–2020	Respiratory infection	LungVITamin D and OmegA-3 Trial	Prevention, treatment	2000 IU daily	Active	USA	NCT01728571
7	2018–2020	Respiratory infection	Maintain respiratory muscle function and reduce pneumonia risk in cancer patients	Prevention	Calcitriol, 0.25 µg/daily	Active	USA	NCT03469271
8	2018–2023	Respiratory infection	Vitamin D in the prevention of viral-induced asthma in preschoolers	Prevention	100,000 IU at baseline, followed by 400 IU daily	Active	Canada	NCT03365687
9	2019–2020	Respiratory infection	Effect of vitamin D supplementation on improvement of pneumonic Children	Treatment	A single 100,000 IU	Active	Egypt	NCT04244474
10	2013–2023	Autoimmune disease	Efficacy of cholecalciferol (vitamin D3) for delaying the diagnosis of MS after a clinically isolated syndrome	Treatment	One 100,000 IU/14 days	Active	France	NCT01817166
11	2018–2020	Autoimmune disease	longitudinal effect of vitamin D3 replacement on cognitive performance and MRI markers in multiple sclerosis patients	Treatment	50,000 IU weekly	Active	Lebanon	NCT03610139
12	2011–2022	Autoimmune disease	Vitamin D and fish oil for autoimmune disease, inflammation and knee pain	Prevention, treatment	2000 IU daily	Active	USA	NCT01351805
13	2019–2023	Neurological disorder	High-dose vitamin D supplements in older adults	Prevention	4000 IU daily	Active	USA	NCT03613116
14	2017–2023	Neurological disorder	High-dose vitamin D induction in optic neuritis	Treatment	5 days of 50,000 IU daily, followed by 10,000 IU daily	Active	USA	NCT03302585
15	2016–2020	Neurological disorder	Vitamin D deficiency and dysautonomia	Prevention	Not available	Active	USA	NCT03032328

##### Vitamin D has antimalarial and anti-SLE function

Previous in vitro studies have shown that vitamin D has antiplasmodial activity through VDR dependent and independent pathways [67]. The onset and progression of malaria has been shown to involve excessive Th1 cell responses, reduction of Th2 response, and dysfunction of Treg cells, all of which can be limited by the action of vitamin D. Furthermore, vitamin D is known to inhibit the synthesis of key pro-inflammatory cytokines involved in the development of fatal cerebral malaria such as IFN-γ and TNF-α [67]. Vitamin D administered after an acute malarial infection increased the survival of diseased mice by at least 15 days compared to non-treated mice through inhibition of IFN-γ and TNF-α synthesis [68].

Low serum levels of 25(OH)D have been associated with TLR-driven amplification of autoimmunity and disease severity in SLE [69]. Hydroxychloroquine (HCQ, Plaquenil), a less toxic derivative of choloroquine, used in medical management of malaria and SLE by inhibiting TLR-driven immune responses, has been demonstrated to have both anti-SARS-CoV and anti-SARS-CoV-2 activities in vitro  [70]. Now, hydroxychloroquine is in ongoing clinical trials to assess its effect on SARS-CoV-2-infected patients and was reported to be significantly associated with reduction of viral load and improvement of symptoms in COVID-19 patients [71]. The exact mechanism of hydroxychloroquine is still not fully understood, but it might inhibit viral entry and replication by exerting endosomal acidification, cytotoxicity, and suppression of inflammation [72]. Considering the similarity in mechanisms of anti-viral infection and anti-inflammation between vitamin D and hydroxychloroquine, we hypothesize that vitamin D supplementation might be applicable to improve clinical symptoms in COVID-19. There are ongoing clinical trials of vitamin D only or combining vitamin D with hydroxychloroquine in the treatment of COVID-19 (Studies 1–4, Table 1).

## Vitamin D might act as an immunosuppressant by inhibiting Cytokine Release Syndrome in COVID-19

It is increasingly recognized that localized synthesis of 1,25(OH)_2_D_3_ rather than systemic production is responsible for many of the immune effects of vitamin D in respiratory diseases [73]. Extra-renal expression of CYP27B1 has been found in alveolar macrophages, dendritic cells, lymphocytes, and epithelia, which form 1,25(OH)_2_D_3_ locally to act in an autocrine or paracrine fashion to modulate cell proliferation, cell differentiation and inflammation [62, 73]. At the beginning stages of acute inflammation (elicitation phase), vitamin D inhibits proliferation of Th1 and Th17 cells and the abnormal release of their cytokines (IFN-γ, TNF-α, IL-1, IL-2, IL12, IL-23 and IL-17, IL-21) [74]. During the resolution phase of inflammation, vitamin D-mediated differentiation of Th2 cells and release of their cytokines (IL-4 and IL-10) are important to avoid organ damage through an excessive immune response. Mechanistically, vitamin D treatment can decrease mRNA expression of IFN-β and interferon-stimulated genes in respiratory syncytial virus (RSV) [63]. Downregulation of pro-inflammatory cytokines is another important mechanism by which vitamin D exerts its immunomodulatory effects in the pulmonary infection. It has been reported to reduce downstream targets of TNF-α, directly modulate NF-κB activity in immune cells and indirectly inhibit NF-κB signaling by upregulating the expression of insulin-like growth factor binding protein-3 (IGFBP-3) [75]. Additionally, vitamin D can decrease expression of IL-6 through MAPKs/P38 signaling pathway [76]. IL-6 was thought to play a key role in the cytokine storm associated with serious adverse outcomes and lower NK cell numbers in patients infected with SARS-CoV-2 pneumonia [48]. Anti-IL6 treatment is in clinical trial for severe respiratory failure in COVID-19 (ClinicalTrials.gov Identifier: NCT04322773). In addition, there are ongoing clinical trials of vitamin D in prevention and treatment of autoimmune diseases by utilizing its immunomodulatory function (Studies 10–12, Table 1). Considering its role as a strong immune-suppressor, vitamin D supplementation might help inhibit abnormal immune response and cytokine storm in COVID-19.

## Vitamin D might prevent loss of neural sensation in COVID-19 by stimulating expression of neurotrophins like Nerve Growth Factor

Some COVID-19 patients have neurological symptoms—loss of taste and smell, headaches, etc. How SARS-CoV-2 affects the nervous system has not been well studied because few autopsies of these patients have been done due to the highly contagious virus. Previous evidence from studies of SARS-CoV have demonstrated the presence of SARS-CoV in neurons of the brain from SARS patients [77], and suggest that the coronavirus might target the nervous system through the olfactory bulb [78]. It’s possible that SARS-CoV-2 can infect nerve cells, particularly neurons in the nervous system and cause nerve damage leading to neurological symptoms in COVID-19 [79].

Previous studies have revealed that vitamin D has a potent neuroprotective effect through several independent mechanisms [80]. The neuroprotective action of vitamin D is associated with regulation of neurotrophins, which are important for survival, differentiation, and maintenance of nerve cells in both peripheral and central nervous systems [81]. Vitamin D stimulates expression of NGF, brain-derived neurotrophic factor (BDNF), neurotrophin-3 (NT3), glial neurotrophic factor, and also neurotrophin receptor p75^NTR^ in neurons, glial cells, and schwann cells. Neurotrophin induction underlies the neuroprotective effect of vitamin D in brain ischemia and neurodegenerative disorders. For example, vitamin D can decrease the progression of Alzheimer’s disease through the induction of NGF [81].

The immunomodulating properties of vitamin D represent another mechanism of its activity as an immunosuppressant to protect neurons [82]. Vitamin D has been reported to promote the migration and differentiation of oligodendrocyte progenitors and enhance remyelination of neurons to improve neurotransmission in diseased rats [83]. There are ongoing clinical trials of vitamin D in prevention and treatment of neurological diseases by utilizing neuroprotective effects (Studies 13–15, Table 1). Our hypothesis is that vitamin D might improve anosmia-like symptoms and prevent neurological complications in COVID-19.

## Discussion

### Yin and Yang of vitamin D application

Low levels of 25(OH)D are common and have been associated with a variety of disease states, including susceptibility to respiratory infections [84]. Clinical data have shown that vitamin D has a protective effect against respiratory tract infections, and meta-analyses support the inverse relationship of 25(OH)D levels and the incidence of influenza [85, 86]. However, due to the trial design, patient selection, etc., vitamin D supplementation and its application in the prevention and treatment of human diseases may not show a benefit [87]. One way to address this concern is to recognize that decreased levels of 1,25(OH)_2_D_3_ with increased age is a major factor of weakened immune response with age (immunosenescence). In a previous study in Norway patients, 1,25(OH)_2_D_3_ was found to decrease from 140 pmol/l for those aged 20–39 years to 98 pmol/l for those > 80 years, despite an increase in serum 25(OH)D from 24 ng/ml (59.9 nmol/L) for those 20–39 years to 27 ng/ml (67.4 nmol/L) for those > 80 years [88]. Thus, vitamin D levels should not be solely dependent on 25(OH)D levels but should include the measurement of levels of 1,25(OH)_2_D_3_ as well. The concentration of 1,25(OH)_2_D_3_ should be considered on several levels: 1) 25(OH)D substrate level; 2) renal expression of 1α-hydroxylase or CYP27B1; 3) extra-renal expression of CYP27B1 and 4) local expression of 25-hydroxyvitamin D-24α-hydroxylase [19]. Vitamin D needs CYP27B1 to generate the biologically active 1,25(OH)_2_D_3_ in renal or extra-renal tissues. In our recent study of acute myeloid leukemia (AML) cells, we observed significantly lower levels of CYP27B1 proteins within bone marrow (BM) cells when compared to non-AML controls [89]. In similar studies of colorectal cancer patients, expression and activity of CYP27B1 in cancer cells were found lower than in normal cells [34].

Additionally, we recently demonstrated that serum levels of 1,25(OH)_2_D_3_ were significantly lower in non-survivors compared to survivors among sepsis patients, and that complex mechanisms underlie low 1,25(OH)_2_D_3_ in critically ill patients [27].

### Recommendation of vitamin D supplementation

Our hypothesis is that vitamin D supplementation can reduce the risk of COVID-19 incidence, symptoms, and severity. It should be investigated in a well-designed clinical trial as vitamin D is in general safe and there is a large body of data supporting the use of higher doses of Vitamin D3 in the setting of vitamin D deficiency.

We propose that COVID-19 patients have vitamin D levels (25(OH)D and 1,25(OH)_2_D_3_) monitored and replaced to meet the baseline requirement.

Guidelines recommended a target level of 30–40 ng/mL (75–100 nmol/L) for patients with risk of fractures, falls, autoimmune conditions, and cancers [90]. The Endocrine Society recommends vitamin D supplementation of < 10,000 IU daily with a target 25(OH)D concentration of 30 ng/ml (75 nmol/L) or higher for patients or anyone with chronic disease [91]. The U.S. Institute of Medicine considers a quantity below 4000 IU/day to be safe [92]. The most common forms of vitamin D for supplementation are cholecalciferol (Vitamin D_3_) and ergocalciferol (Vitamin D_2_), precursors of 1,25(OH)_2_D_3_, although administration of calcitriol is limited because of the potential hypercalcemia-related complications.

In a previous study of vitamin D supplementation in those with SLE, 50,000 IU of vitamin D weekly for 128 weeks were prescribed for those patients with levels less than 40 ng/ml (100 nmol/L) and the results suggest that high doses of vitamin D can reduce disease activity [93]. It is important to mention that although cholecalciferol or ergocalciferol is safer compared to 1,25(OH)_2_D_3_, high doses of vitamin D supplementation may still cause systemic hypercalcemia, another major obstacle of vitamin D therapy. The concentrations of 1,25(OH)_2_D_3_ required to induce differentiation in vitro are typically in the range of 10–100 nM (nM); however, a serum level of such a concentration would result in hypercalcemia in humans as the typical concentration of 1,25(OH)_2_D_3_ is around 0.1 nM. One potential strategy is to develop a tissue-specific approach to deliver therapeutic levels of 1,25(OH)_2_D_3_ locally in the absence of hypercalcemia [94]. There is an inverse correlation of age and CYP27B1 expression in human parathyroid glands, which may be due to increased levels of intact PTH with age [95]. A similar approach has also been demonstrated to be effective in treating leukemia mouse models, by delivering ectopic CYP27B1 to local tissues such as bone marrow cells to compensate the deficiency or incapable generation of CYP27B1 as shown in our recent study [89]. Our data suggest that engraftment of ectopic CYP27B1 in leukemia bone marrow is critical for both reduction of leukemia blasts and protection of host microenvironment for healthy hematopoietic stem cells’ recovery without systemic hypercalcemia. Finally, increased local synthesis of high dose 1,25(OH)_2_D_3_ can be achieved by focused stimulation of locally regulated CYP27B1 in inflammatory cells to exert autocrine/paracrine actions [96].

## Conclusion

**Fig. 4:**
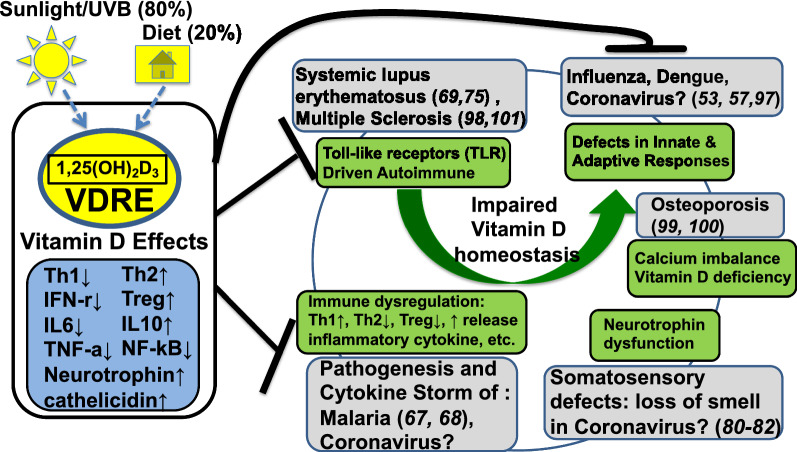
Schematic overview of vitamin D treatment of human diseases. Vitamin D deficiency is common. Low levels of vitamin D have been reported to be involved in the pathogenesis of various human diseases including autoimmune diseases like SLE, cytokine release syndrome, somatosensory defects, osteoporosis, and infections like influenza and dengue. Vitamin D supplementation is required for maintaining a balanced immune system to fight pathogens and prevent autoimmune diseases. The green box indicates the mechanism of the diseases. The blue box indicates immunomodulatory effects of vitamin D

There is a plethora of evidence that vitamin D is required for normal immune function for fighting pathogens and preventing autoimmune diseases [75, 97–101] (Fig. 4). Published clinical trial data also demonstrated the potential of vitamin D supplementation to prevent acute respiratory infection through modulating the innate immune response [29, 66] and boosting antibody production after vaccination [30, 102]. While there are various outcomes regarding the benefit of vitamin D supplementation in the clinical research, the clear association between low levels of 25(OH)D or 1,25(OH)_2_D_3_ and pathogenesis of a wide variety of infectious and autoimmune diseases is warranted for further investigation of vitamin D supplementation (readily available and affordable) as a potential agent to prevent viral infection and improve the survival outcome. Hence, we hypothesize that vitamin D supplementation will help COVID-19 patients maintain sufficient serum levels of vitamin D as guideline recommended [91, 103], allowing inflammation-upregulated CYP27B1 in respiratory epithelia and immune cells to generate a local high dose of 1,25(OH)_2_D_3_ to inhibit CRS and disease progression. Serum calcium level should be monitored and if significantly elevated, it can be mitigated by hydration or a dose adjustment of the vitamin D supplement. There is no current Food and Drug Administration (FDA)-approved vitamin D treatment for diseases. However, section 101.72 of FDA (revised as of April 1, 2019) mentioned that adequate calcium and vitamin D may reduce the risk of osteoporosis. In the era of overwhelming COVID-19 disease burden, the risk and benefit of such trials (Additional file 1: Table S1: 21 ongoing vitamin D clinical trials for COVID-19 with detailed information, as of July 2nd, 2020) is highly favorable while no specific therapy or vaccine is available for COVID-19 currently.

## Supplementary information

**Additional file 1: Table S1.** Ongoing Clinical Trials as of July 2nd, 2020, of vitamin D in the Prevention and Treatment of COVID-19.

## Data Availability

Not applicable

## Abbreviations

COVID-19: Coronavirus disease 2019
SARS-CoV-2: Severe acute respiratory syndrome coronavirus-2
CRS: Cytokine release syndrome
ARDS: Acute respiratory distress syndrome
SLE: Systemic lupus erythematosus
NF-kB: Nuclear factor kappa B
IL: Interleukin
TNF: Tumor necrosis factor
NGF: Nerve growth factor
UVB: Ultraviolet B
25(OH)D: 25-Hydroxyvitamin D
CYP27B1: 1α-Hydroxylase
1,25(OH)_2_D_3_: 1α,25-Dihydroxyvitamin D3
VDR: Vitamin D receptor
VDRE: Vitamin D response element
PTH: Parathyroid hormone
FGF 23: Fibroblast growth factor 23
TLRs: Toll-like receptors
Treg: Regulatory T cells
Th: T-helper
NK: Natural killer cells
HIV: Human immunodeficiency virus
EBV: Epstein Barr virus
HCV: Hepatitis C virus
Rota: Rotavirus
IFNr: Interferon gamma
IU: International units
Rota: Rotavirus
RSV: Respiratory syncytial virus
IGFBP-3: Insulin-like growth factor binding protein-3
nM: Nanomolar
